# Semiquantative Visual Assessment of Sub-solid Pulmonary Nodules ≦3 cm in Differentiation of Lung Adenocarcinoma Spectrum

**DOI:** 10.1038/s41598-017-16042-9

**Published:** 2017-11-17

**Authors:** Fu-Zong Wu, Po-An Chen, Carol C. Wu, Pei-Lun Kuo, Shu-Ping Tsao, Chu-Chun Chien, En-Kuei Tang, Ming-Ting Wu

**Affiliations:** 1 Department of Radiology, Kaohsiung Veterans General Hospital, Kaohsiung, Taiwan; 2 Faculty of Medicine, School of Medicine, National Yang Ming University, Taipei, Taiwan; 3 Institute of Clinical Medicine, National Yang Ming University, Taipei, Taiwan; 4 School of Medicine, College of Medicine, Kaohsiung Medical University, Kaohsiung, Taiwan; 5 Department of Radiology, University of Texas MD Anderson Cancer Center, Houston, TX USA; 6 Department of Pathology and Laboratory Medicine, Kaohsiung Veterans General Hospital, Kaohsiung, Taiwan; 7 Department of Pathology, Kaohsiung Municipal Ta-Tung Hospital, Kaohsiung, Taiwan; 8 Department of Surgery, Kaohsiung Veterans General Hospital, Kaohsiung, Taiwan

## Abstract

We aimed to analyze CT features of persistent subsolid nodules (SSN) ≦3 cm diagnosed pathologically as adenocarcinoma spectrum to investigate whether parameters enable distinction between invasive pulmonary adenocarcinomas (IPAs) and pre-invasive lesions. A total of 129 patients with 141 SSNs confirmed with surgically pathologic proof were retrospectively reviewed. Of 141 SSNs, there were 57 pure ground-glass nodules (GGNs), 22 heterogeneous GGNs, and 62 part-solid nodules. SSN subclassification showed a significant linear trend with invasive degree of the adenocarcinoma spectrum (pure GGNs 7%; heterogeneous GGNs 36.4%; part-solid nodules 85.5%, *P* for trend <0.0001). For IPA detection in 141 SSNs, a solid part of ≧3 mm was the most specificity (sensitivity, 76.9%; specificity, 94.7%), followed by air-bronchogram sign (sensitivity, 53.8%; specificity, 89.5%), SSN subclassification (sensitivity, 81.5%; specificity, 88.2%), and a lesion size ≧12 mm (sensitivity, 84.6%; specificity, 76.3%). For IPA detection in 79 pure or heterogeneous GGNs, the heterogeneous GGN sign was the most useful finding, with most specificity (sensitivity, 66.7%; specificity, 79.1%), followed by CT attenuation (HU) of ≧−493 (sensitivity, 75%; specificity, 74.6%) and a lesion size ≧10 mm (sensitivity, 83.3%; specificity, 70.1%). In conclusion, this simple combined visual and semiquantitative analysis of CT features helps distinguish IPAs from pre-invasive lesions.

## Introduction

With the introduction of low-dose computed tomography (LDCT) for lung cancer screening in recent years, persistent subsolid nodules (SSNs) have played an important role in highly association with lung adenocarcinomas^1–4^. This new classification addresses these new concepts of invasiveness degree in the adenocarcinoma spectrum, such as atypical adenomatous hyperplasia (AAH), adenocarcinoma *in situ* (AIS), minimally invasive adenocarcinoma (MIA) and invasive pulmonary adenocarcinoma (IPA) according to the 2011 International Association for the Study of Lung Cancer, the American Thoracic Society, and the European Respiratory Society (IASLC/ATS/ERS) classification^5^. Because IPA lesions have clinically more aggressive behavior than their pre-invasive or minimally invasive precursors, early recognition of the invasive characteristics based on computed tomography (CT) features would be clinically important and could provide guidance to the clinical decision making. The new concept of the pulmonary adenocarcinoma spectrum in differentiation IPA from per-invasive/minimally invasive lesions would help determine the optimal operation time and select appropriate candidates for sublobar resection in the era of LDCT lung cancer screening in Asian. Recent studies have investigated the radiologic-pathologic correlation of the adenocarcinoma spectrum more clearly, and have the potential ability to differentiate pre-invasive/minimally lesions from IPA through segmentation software for texture analysis and measurement of the volume, mass and diameter of SSNs^6–12^. But this software was not yet available for clinical routine practice for SSNs^13^.

Recent studies have demonstrated that the solid/invasive component is the major determinant of prognosis in prediction of invasive degree of the lung adenocarcinoma spectrum^14–16^. However, the criteria and definition of a solid component are still controversial (e.g., volumetric/diameter measurement, lung/mediastinal windows, section thickness due to partial volume effect). A large prospective cohort study by Kakinuma *et al*. provides some guidance with the new SSN subclassification system, distinguishing pure groundglass nodules (GGNs), heterogeneous GGNs, and part-solid nodules^17,18^. In this study, we want to investigate the association between characteristic CT features and the pathologic invasiveness of the lung adenocarcinoma spectrum based on the novel SSN classification system.

## Results

### Demographics and clinical characteristics

A total of 129 subjects with 141 SSNs were enrolled and summarize in Table 1. There were 65 subjects with pre-invasive/minimally invasive lesions and 64 subjects with IPAs. There were no significant differences in sex ratio and the frequency of multiple primary lung cancer (MPLC) percentages among these two groups. Compared with the pre-invasive/minimally invasive group, there was significantly higher age and a high percentage of Lung Imaging Reporting and Data System (Lung-RADS) distribution classified as 4 in the IPA group.

**Table 1:** Clinical Characteristics of 129 subjects in reference to IPA lesions or pre-invasive/minimally invasive lesions.

Characteristics	Pre-invasive/minimally invasive (N = 65)	IPA (N = 64)	*P*-Value
Age (year)	56.33 ± 9.44	62.48 ± 10.47	0.001^*^
Male-to female ratio			0.431^**^
No. of male	14	12	
No. of female	51	52	
The percentage of MPLC	18 (27.7%)	21 (20.9%)	0. 330^**^
Lung-RADS distribution			<0.0001^***^
1	0	0	
2	54 (83.1%)	10 (15.6%)	
3	5 (7.7%)	3 (4.7%)	
4	6 (9.2%)	51 (79.7%)	

^*^Using independent t-test for continuous variables; ^**^Using Chi-square test for categorical variables; ^***^Using Fisher’s exact test for categorical variables. Abbreviations: IPA: invasive pulmonary adenocarcinoma; MPLC: multiple primary lung cancer; Lung-RADS: The ACR Lung Imaging Reporting and Data System.

For mean lesion size, IPA lesions were significantly greater in comparison to pre-invasive/minimally invasive lesions (20.31 ± 9.26 mm vs. 12.27 ± 2.90 mm, *P* = 0.013). For solid part, IPA lesions were significantly greater in comparison to pre-invasive/minimally invasive lesions show in Supplement Table 1 (10.27 ± 5.89 mm vs. 4.50 ± 2.73 mm, *P* = 0.006).

The characteristics of the 129 subjects with 141 SSNs according to the new subclassification system were shown in Table 2. Of 141 SSNs, 57 were pure GGNs, 22 were heterogeneous GGNs, and 62 were part-solid nodule according to this subclassification. Surgical histologic examinations revealed AAH in 8 patients, AIS in 19 patients, MIA in 49 patients, and IPA in 65 patients. The proportion of IPA was 7% (N = 4) in pure GGNs group, 36.4% (N = 8) in the heterogeneous GGNs group, and 85.5% (N = 53) in the part-solid nodules group. The percentage of IPA and lesion size of SSNs were positively associated with SSN subclassification (*P* for trend <0.0001). The lesion size, solid part, air-bronchogram sign, cyst-like airspace, CT attenuation expressed in Hounsfield Units (HU, min/max/mean) and Lung-RADS distribution, which these descriptive characteristics and quantitative parameters of the 141 SSNs across three subcategories of SSNs in this study are shown in Table 2.

**Table 2:** Imaging features and pathologic results of 141 subsolid nodules according to IASLC/ATS/ERS International Multidisciplinary Classification based on SSN subclassfication.

	Pure GGN (N = 57)	Heterogeneous GGN (N = 22)	Part-solid nodule (N = 62)	*P*-value	*P*-value for Trend
Lesion size	9.081 ± 4.17	13.841 ± 5.98	19.145 ± 9.0818	<0.0001^****^	<0.0001
Solid part	N/A	N/A	9.435 ± 5.8991	N/A	N/A
Air-bronchogram	0	4	39	<0.0001^***^	N/A
Cyst-like airspace	1	0	4	0.09^***^	N/A
HU (mean)	−592.01 ± 113.55	−435.27 ± 102.49	N/A	<0.0001^*^	N/A
HU (max)	−526.87 ± 123.81	−327.545 ± 125.56	N/A	<0.0001^*^	N/A
HU (min)	−676.368 ± 129.069	−578.59 ± 198.085	N/A	0.002^*^	N/A
IPA lesions	4 (7.0%)	8 (36.4%)	53 (85.5%)	<0.0001^****^	<0.0001
Adenocarcinoma spectrum				<0.0001^***^	N/A
AAH	8	0	0		
AIS	17	2	0		
MIA	28	12	9		
IPA	4	8	53		
Lung-RADS distribution				<0.0001^***^	N/A
1	0	0	0		
2	55	21	0		
3	2	1	5		
4	0	0	57		

^*^Using independent t-test for continuous variables; ^**^Using Chi-square test for categorical variables; ^***^Using Fisher’s exact test for categorical variables. ^****^Using one-way ANOVA to compare three groups.

Abbreviations: AAH: atypical adenomatous hyperplasia; AIS: adenocarcinoma *in situ*; HU: Hounsfield unit; IPA: invasive pulmonary adenocarcinoma; MIA: minimally invasive adenocarcinoma; GGN: groundglass nodule; SSN = subsolid nodule. ANOVA: analysis of variance.

### CT imaging features of 141 SSNs

For 141 SSNs, IPA lesions were more likely to have air-bronchogram, cystic-like airspace, the percentage of part-solid nodule (according to subclassification in this study) in comparison to pre-invasive/minimally invasive lesions shown in Supplement Table 2. For mean lesion size, IPA lesions were significantly greater in comparison to pre-invasive/minimally invasive lesions (19.42 ± 8.99 mm vs. 9.82 ± 4.27 mm, *P* < 0.0001). For solid part, IPA lesions were significantly greater in comparison to pre-invasive/minimally invasive lesions (8.37 ± 6.66 mm vs. 0.57 ± 1.73 mm, *P* < 0.0001). The results of the univariate logistic regression analysis of 141 SSNs in distinguishing pre-invasive/minimally invasive lesions from IPA lesions are illustrated in Supplement Table 3. This result indicates that age, lesion size, solid part, part-solid nodules based on SSN subclassification, and air-bronchogram were significantly associated with IPA lesions shown in Supplement Table 3 (*P* < 0.0001, respectively). For diagnostic tests, sensitivity, specificity, positive predictive value (PPV), negative predictive value (NPV), positive likelihood ratio (LR+), and negative likelihood ratio (LR−) are usually used as performance measures.

The cutoffs and the diagnostic performances of potential predictive parameters in distinguishing the pre-invasive/minimally invasive lesions and IPA lesions as determined by Receiver operating characteristic (ROC) curves analyses are summarized in Table 3 and Supplement Fig. 1. The optimal cut-off value for lesion size in differentiating pre-invasive/minimally invasive lesions from IPA lesions was ≧12 mm with a sensitivity of 84.60% and specificity of 76.30% (PPV = 75.30%; NPV = 85.30%). The optimal cut-off value for solid part size in differentiating pre-invasive/minimally invasive lesions from IPA lesions was ≧3 mm with a sensitivity of 76.9% and specificity of 94.70% (PPV = 92.60%; NPV = 82.80%). Among these potential predictive parameters, lesion size was the most sensitive sign. However, the solid part was the most specific sign.

**Table 3:** The diagnostic performances of the cutoff values for the different variables in distinguishing pre-invasive/minimally invasive lesions and IPA lesions in 141 subsolid nodules.

Characteristics	Cutoff value	AUC	Sensitivity	Specificity	PPV	NPV	Positive LR	Negative LR
Lesion size (mm)	≧12 mm	0.891	84.60%	76.30%	75.30%	85.30%	3.57	0.20
Solid part (mm)	≧3 mm	0.881	76.90%	94.70%	92.60%	82.80%	14.62	0.24
SSN subclassfication	Part-solid nodule	0.886	81.50%	88.20%	85.50%	84.80%	6.89	0.21
Air-bronchogram	Air-bronchogram (+)	0.717	53.80%	89.50%	81.40	69.40	5.12	0.52

Abbreviations: AUC: area under the curve; IPA: invasive pulmonary adenocarcinoma; SSN = subsolid nodule.

### CT imaging features of 79 pure or heterogeneous GGNs

For IPA lesions, the morphologic characteristics of nodules regarding the percentage of heterogeneous GGNs were significantly higher than pre-invasive/minimally invasive lesions (*P* = 0.002). For mean lesion size, IPA lesions were significantly larger in comparison to pre-invasive/minimally invasive lesions (15.50 ± 6.62 mm vs. 9.49 ± 4.33, *P* < 0.001). For CT attenuations, IPA lesions were significantly more dense in comparison to pre-invasive/minimally invasive lesions (HU^mean^ −477.67 ± 132.59 vs. −561.03 ± 127.24, *P* = 0.041; HU^max^ −357.41 ± 171.39 vs. −491.77 ± 141.06, *P* = 0.004; HU^min^ −574.25 ± 107.40 vs. −662.55 ± 130.75, *P* = 0.030). There were no significant differences in the morphologic characteristics of air-bronchogram and cyst-like airspace sign between IPA and pre-invasive/minimally invasive lesions shown in Table 4.

**Table 4:** CT imaging features of pre-invasive/minimally invasive lesions and IPA lesions in 79 pure or heterogeneous GGNs.

Characteristics	Pre-invasive/minimally invasive (N = 67)	IPA (N = 12)	*P*-Value
Lesion size (mm)	9.49 ± 4.33	15.50 ± 6.62	<0.001^*^
SSN subclassfication			0.002^***^
Pure GGN	53	4	
Heterogeneous GGN	14	8	
Air-bronchogram	3 (4.4%)	1 (8.3%)	0.383^***^
Cyst-like airspace	0 (0%)	1 (8.3%)	0.152^***^
HU (mean)	−561.03 ± 127.24	−477.67 ± 132.59	0.041^*^
HU (max)	−491.77 ± 141.06	−357.41 ± 171.39	0.004^*^
HU (min)	−662.55 ± 130.75	−574.25 ± 107.40	0.030^*^

^*^Using independent t-test for continuous variables; ^**^Using Chi-square test for categorical variables; ^***^Using Fisher’s exact test for categorical variables.

Abbreviations: GGN: groundglass nodule; HU: Hounsfield unit; IPA: invasive pulmonary adenocarcinoma.

The results of the univariate logistic regression analysis of 79 pure or heterogeneous GGNs in distinguishing pre-invasive/minimally invasive lesions from IPA lesions are illustrated in Supplement Table 4. This result indicates that lesion size, heterogeneous GGNs and CT attenuation were significantly associated with IPA lesions (*P* = 0.002; *P* = 0.003; *P* = 0.048, respectively). The cutoffs and the diagnostic performances of potential predictive parameters in distinguishing the pre-invasive/minimally invasive lesions and IPA lesions as determined by ROC analyses are summarized in Table 5 and Supplement Fig. 2. The optimal cut-off value for lesion size in differentiating pre-invasive/minimally invasive lesions from IPA lesions was ≧10 mm with a sensitivity of 83.30% and a specificity of 70.10% (PPV = 33.30%; NPV = 95.90%). The optimal cut-off value for SSN subclassification in differentiating pre-invasive/minimally invasive lesions from IPA lesions was heterogeneous GGN with a sensitivity of 66.70% and specificity of 79.10% (PPV = 36.40%; NPV = 93.00%). The optimal cut-off value for CT attenuation in differentiating pre-invasive/minimally invasive lesions from IPA lesions was HU ≧−493 with a sensitivity of 75% and specificity of 74.60% (PPV = 34.60%; NPV = 94.30%). Among these potential predictive parameters, lesion size was the most sensitive sign. However, the heterogeneous GGN sign was the most specific sign.

**Table 5:** The diagnostic performances of the cutoff values for the different variables in distinguishing pre-invasive/minimally invasive lesions and IPA lesions in 79 pure or heterogeneous GGNs.

Characteristics	Cutoff value	AUC	Sensitivity	Specificity	PPV	NPV	Positive LR	Negative LR
Lesion size (mm)	≧10 mm	0.810	83.30%	70.10%	33.30%	95.90%	2.79	0.24
SSN subclassfication	Heterogeneous GGN	0.729	66.70%	79.10%	36.40%	93.00%	3.19	0.42
HU (mean)	≧−493	0.693	75.00%	74.60%	34.60%	94.30%	2.96	0.33

Abbreviations: AUC: the area under the curve; GGN: groundglass nodule; HU: Hounsfield unit; IPA: invasive pulmonary adenocarcinoma; SSN: subsolid nodule.

## Discussion

**Figure 1:**
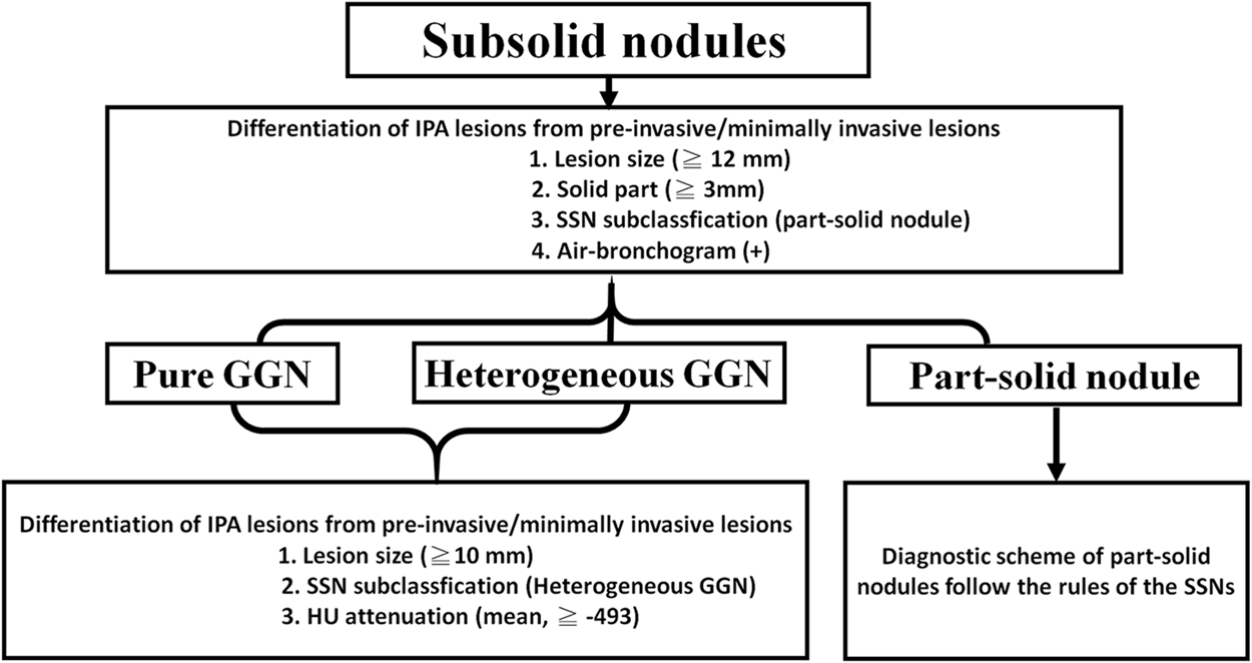
First, to determine the radiologic features of SSNs according to the subclassification system into three different categories. Second, the lesion size with cut-off value of ≧10 mm, the type of heterogeneous GGN, and the higher HU value with cut-off value of ≧−493 in the pure and heterogeneous GGNs were the optimal diagnostic threshold for IPA lesions prediction with high NPV, which could help to rule out IPAs. Third, the lesion size with cut-off value of ≧12 mm, the type of part-solid nodule, and the solid part with cut-off value of ≧3 mm, presence of air-bronchogram in all SSNs were the optimal diagnostic threshold for IPA lesions prediction with moderate to high PPV, which could help to rule in IPAs. Fourth, the diagnostic scheme of part-solid nodules followed the rules of the SSNs.

We demonstrated that simple semiquantitative analysis of CT features enables the differentiation of IPA lesions from pre-invasive/minimally invasive lesions that appear as SSNs with size ≦ 3 cm. In this study, we proposed a new algorithm and flowchart through the simple visual semiquantitative analysis. This scheme describes the flowchart of differentiation of IPAs from pre-invasive/minimally invasive lesions using the SSNs subclassification shown in Fig. 1.

First, to determine the radiologic features of SSNs according to the subclassification system into three different categories. Second, the lesion size with cut-off value of ≧10 mm, the type of heterogeneous GGN, and the higher HU value with cut-off value of ≧−493 in the pure and heterogeneous GGNs were the optimal diagnostic threshold for IPA lesions prediction with high NPV, which could help to rule out IPAs. Third, the lesion size with cut-off value of ≧12 mm, the type of part-solid nodule, and the solid part with cut-off value of ≧3 mm, presence of air-bronchogram in all SSNs were the optimal diagnostic threshold for IPA lesions prediction with moderate to high PPV, which could help to rule in IPAs. Fourth, the diagnostic scheme of part-solid nodules followed the rules of the SSNs. In summary, we could use likelihood ratios to analyze a series of tests according to the SSN subclassification setting. We need to calculate the post-test probability using pre-test probability and likelihood ratios (LRs). Once you have specified the pre-test odds, you multiply them by the likelihood ratio. This gives you the post-test odds. We will use post-test odds = pre-test odds × LR1 × LR2 × LR3… × LR_n_. Finally, the post-test probability could be calculated according to the following formula: Post-test probability = Post-test odds/Post-test odds +1.

For pure or heterogeneous GGN, a lesion size cutoff of ≧10 mm has relative low specificity but higher sensitivity for IPA in these settings (PPV = 33.30%; NPV = 95.90%). This test has the disadvantage of relative high false-positive rate.

Therefore, subjects will potentially receive unnecessary follow-up diagnostic or therapeutic procedures. Combined assessment of the other two parameters would improve the specificity. Preliminary data are promising. Further investigation with larger prospective studies is needed to validate the result and to develop the prediction model through different combinations of parameters.

Our study results support the pure GGN with cutoff vale of 10.5 mm and heterogeneous uniformity of nodule texture significantly associated with invasive lesions in the previous study^19^. The results from previous studies have shown that the optimal cut-off value for discrimining pre-invasive lesions from invasive lesions with pure GGOs was 10.5 cm, and the sensitivity was 86.30%, and the specificity was 61.90%^19^. In addition, another study also revealed that a lesion size of less than 10 mm can be a very specific discriminator of pre-invasive lesions form IPA/MIAs with specificity of 100% in pure GGNs^7^. The differentiation of the results may be due to the misclassification of SSNs into GGN or part-solid nodules^20^. However, these study results suggest that in pure or heterogeneous GGNs, a lesion size of less than 10 mm could indicate that pre-invasive or minimally invasive nature of indolent or slowing-growing lung adenocarcinoma spectrum lesions. These findings also support that NCCN management guideline for <20 mm non-solid nodules (recommend annually follow up for <20 mm non-solid nodules) or 2017 Fleischner guidelines for >8 mm GGNs^21,22^. The combination strategy of a period of watchful waiting with annual or eventual follow-up every two years for at least a period of 5 years and biopsy or surgery after confirmed growth would be a safe option to allow early detection of lesion progression and to avoid the potential risks of over-diagnosis and over-treatment in pure or heterogeneous GGNs.

In our study, we could more clarity more clearly the SSNs into three subclassification (pure GGN, heterogeneous GGN and part-solid nodule). The classification provides both a simple and more consistent solution for SSN subclassification, and would make the study result more robust. We also found that the optimal cutoff value for discriminating pre-invasive/minimally invasive lesions from IPA lesions was mean CT attenuation of ≧−493 HU. And thus our results correlated with previous studies in showing that with an optimal cutoff value of ≧−472 HU for differentiation between bronchioalveolar carcinoma and adenocarcinoma based on the formal definition^8,23^.

A lesion size of less than 10 mm, a CT attenuation threshold value of less than −493, and pure GGN subclassfication could indicate the pre-invasive/minimally invasive nature in pure or heterogeneous GGNs because these three parameters have very high NPV in prediction of IPAs.

In our study, there were 53 of 62 (85.5%) IPAs presented as the part-solid nodules. Therefore, we could use this simple visual method based on lung and mediastinal window to identify most of the IPAs appearing as part-solid nodules. For all SSNs, a lesion size cut-off value of ≧12 mm has relative low specificity for IPAs in these settings. The optimal cut-off value for solid part size in differentiating pre-invasive/minimally invasive lesions from IPA lesions was ≧3 mm with a sensitivity of 76.9% and specificity of 94.70%. Among all parameters in IPA prediction, the solid part with cut-off value of ≧3 mm demonstrated the highest specificity with very high positive LR of 14.62. The results from the previous study had also shown that the specificity for IPA prediction ranges from 86% to 96% when the solid portion is measurable in the mediastinal window setting^24^. The solid part with cut-off value of ≧3 mm could accurately indicate the invasive extent in SSNs to differentiate IPA lesions from pre-invasive/minimally invasive lesions.

Our results also demonstrated that the air-bronchogram sign was important CT sign for prediction the pathological invasiveness of SSNs with high specificity. One possible mechanism is the that bronchial or bronchiolar invasion by tumor cell infiltration, which resulting in tortuosity, dilatation, and ectasis of the airways due to subsequent destruction of cartilage or elastic layer^9^. An alternative postulation is that air bronchogram in early IPA was associated with shrinkage of intratumoral fibrosis, which subsequent resulting in abnormal airway’s dilation^25^. And thus our results correlated with these previous studies^9,19,25,26^.

Our study has demonstrated for the first time that the most specific and powerful parameter for predicting IPA was the optimal cut-off diameter of solid part size (≧3 mm) in the mediastinum window, which yielded a sensitivity of 76.9% and specificity of 94.70%, respectively with an area under the curve (AUC) of 0.881. Previous studies revealed that the most powerful parameter for predicting IPA was the diameter of the solid component (≧6.7 mm) in the lung window setting^27^. Although previous studies have demonstrated that the lung window setting had a higher accuracy and better correlation with pathologic specimens than the mediastinal window setting for solid component measurement in SSNs^28^. But this study also showed that interobserver agreement on the nodule classification was found to be slightly lower on the lung window than on mediastinal window setting, but no significant differences between two window settings^28,29^. Besides, it is sometimes difficult in distinguishing a solid part from a vessel in the lung window setting for SSNs.

Our study is the first time to determine optimal cutoff value of solid part in the mediastinal window setting. We believe the simple classification system would provide more consistent solution for SSNs subclassification and solid part measurement. Therefore, it will lead to more robust results.

Implementation of imaging reporting and data system, and incorporation of this subclassification system in further distinguishing IPA and pre-invasive/minimally invasive lesions in persistent SSN would make LDCT lung cancer screening program more efficient and enhance clinical decision to avoid over-diagnosis and over-management of persistent SSNs^30–33^.

The current study has several limitations. First, there was a potential selection bias in our study subjects. In this study we included only pathologically proved SSNs ≦ 3 cm. Second, this was a retrospective study with a relatively small number of subjects. These study results should be further validated in large prospective cohort studies. Third, inter-observer variation is a potential limitation in the pathologic diagnosis of adenocarcinoma spectrum and pathologic invasive component in previous studies have addressed that the issue of fair reproducibility distinguishing invasion from preinvasive lesions in the lung adenocarcinoma spectrum^34^. Nevertheless, we tried to reduce inter-observer and intra-observer variability by consensus double reading of 2 experienced thoracic pathologists.

In SSNs, IPA lesions can be accurately distinguished from pre-invasive/minimally invasive lesions by lesion size (≧12 mm), solid part size (≧3 mm), part-solid nodule based on SSN subclassification, and presence of air-bronchogram sign. Among these parameters in IPA prediction, the solid part with cut-off value of ≧3 mm demonstrated the highest specificity with very high positive LR of 14.62, which could help to rule in IPAs.

In pure and heterogeneous GGNs, the lesion size with cut-off value of ≧10 mm, the type of heterogeneous GGN, and the higher HU value with cut-off value of ≧−493 were the optimal diagnostic threshold for IPA lesions prediction with high NPV, which could help to rule out IPAs. In conclusion, this simple combined visual and semiquantitative analysis of CT features enables help distinguish IPA lesions from pre-invasive/minimally invasive lesions shown as SSNs ≦ 3 cm.

## Methods

### Study cohort

A search of the electronic records and pathology/radiology information systems of our hospital for subjects with pulmonary adenocarcinoma spectrum pathologically surgical diagnosed and manifesting as persistent SSNs ≦ 3 cm on LDCT scan. From May 2012 to October 2016, there was a total of 165 subjects eligible for the pathologic report review. Among them, 36 subjects were excluded due to inappropriate CT images analysis for SSNs analysis (N = 21, section thickness ≧3 mm) or the time period between LDCT scan and operation of more than three months (N = 15). Finally, a total of 129 subjects with 141 SSNs ≦ 3 cm met the inclusion criteria and were reviewed.

### CT image acquisition protocol and CT features analysis

**Figure 2:**
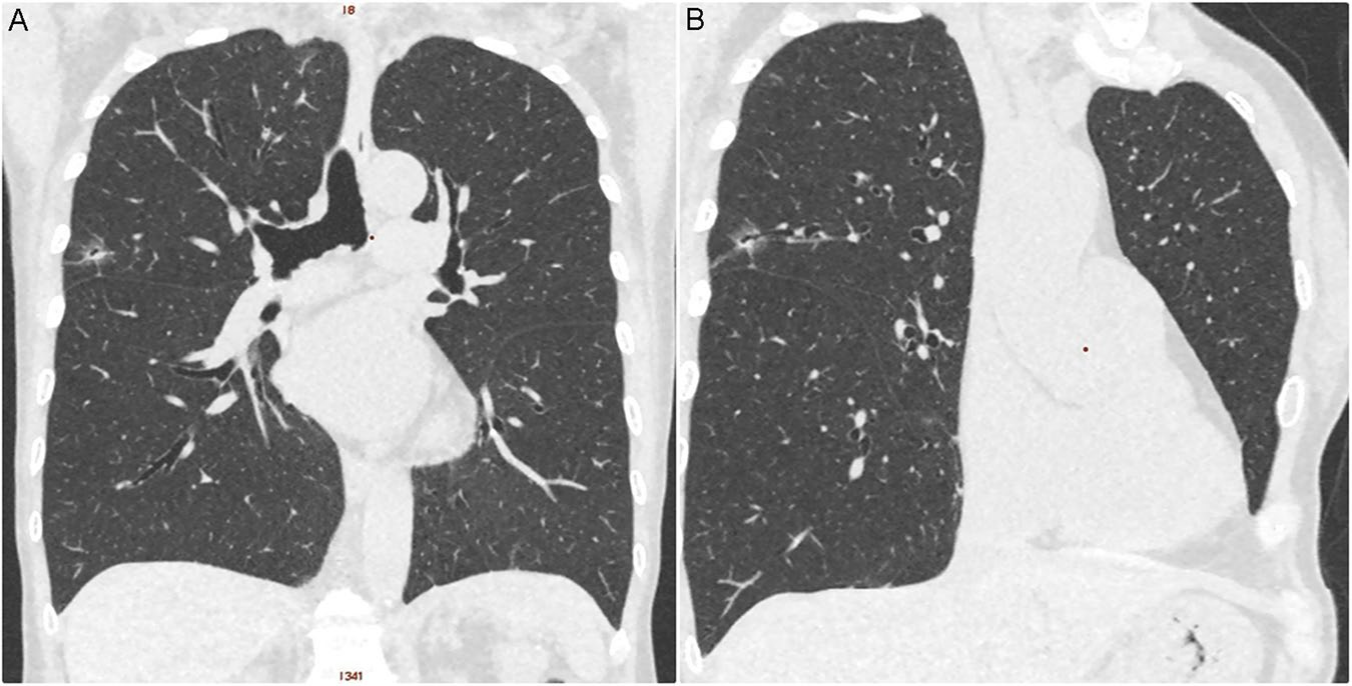
An example of subsolid nodule with an air bronchogram sign according to the SSN subclassification. A 61-year-old woman had a 1.4 cm part-solid nodule in RUL. The (**A**) coronal and (**B**) oblique images showed an internal air bronchogram inside the lesion. The patient underwent video-thoracoscopic wedge resection of RUL. Further pathologic report demonstrated invasive pulmonary adenocarcinoma in RUL, Stage 1. Abbreviations: SSN = subsolid nodule; RUL = right upper lobe.

**Figure 3:**
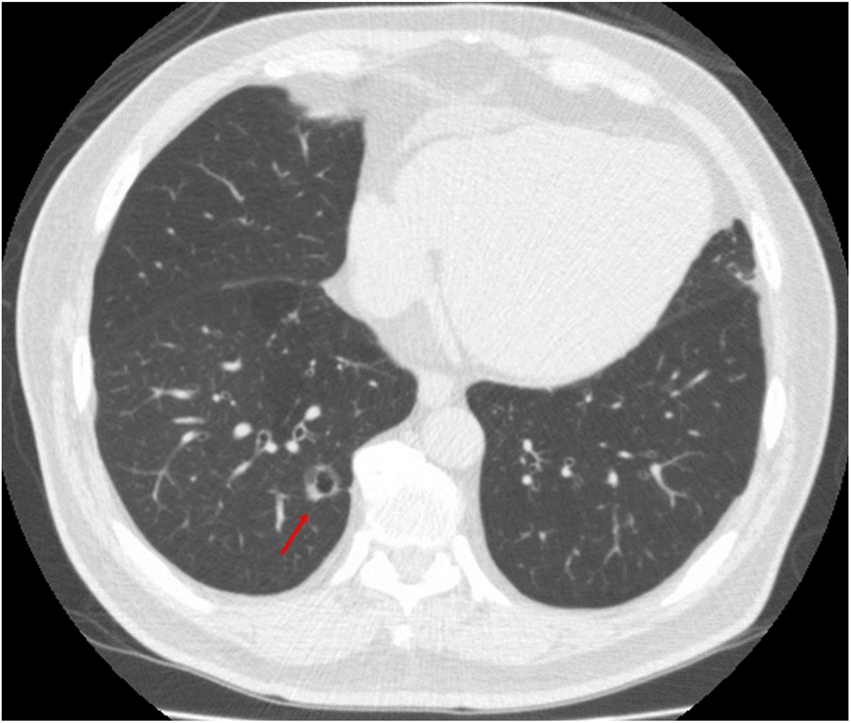
An example of subsolid nodule with abnormal cystic-like airspace according to the SSN subclassification. A 55-year-old man had a 1.1 cm part-solid nodule in RLL. The axial CT image showed an abnormally dilated cystic-like airspace inside the lesion. The patient underwent video-thoracoscopic wedge resection of RLL. Further pathologic report demonstrated invasive pulmonary adenocarcinoma in RLL, Stage 1. Abbreviations: SSN = subsolid nodule; RLL = right lower lobe.

**Figure 4:**
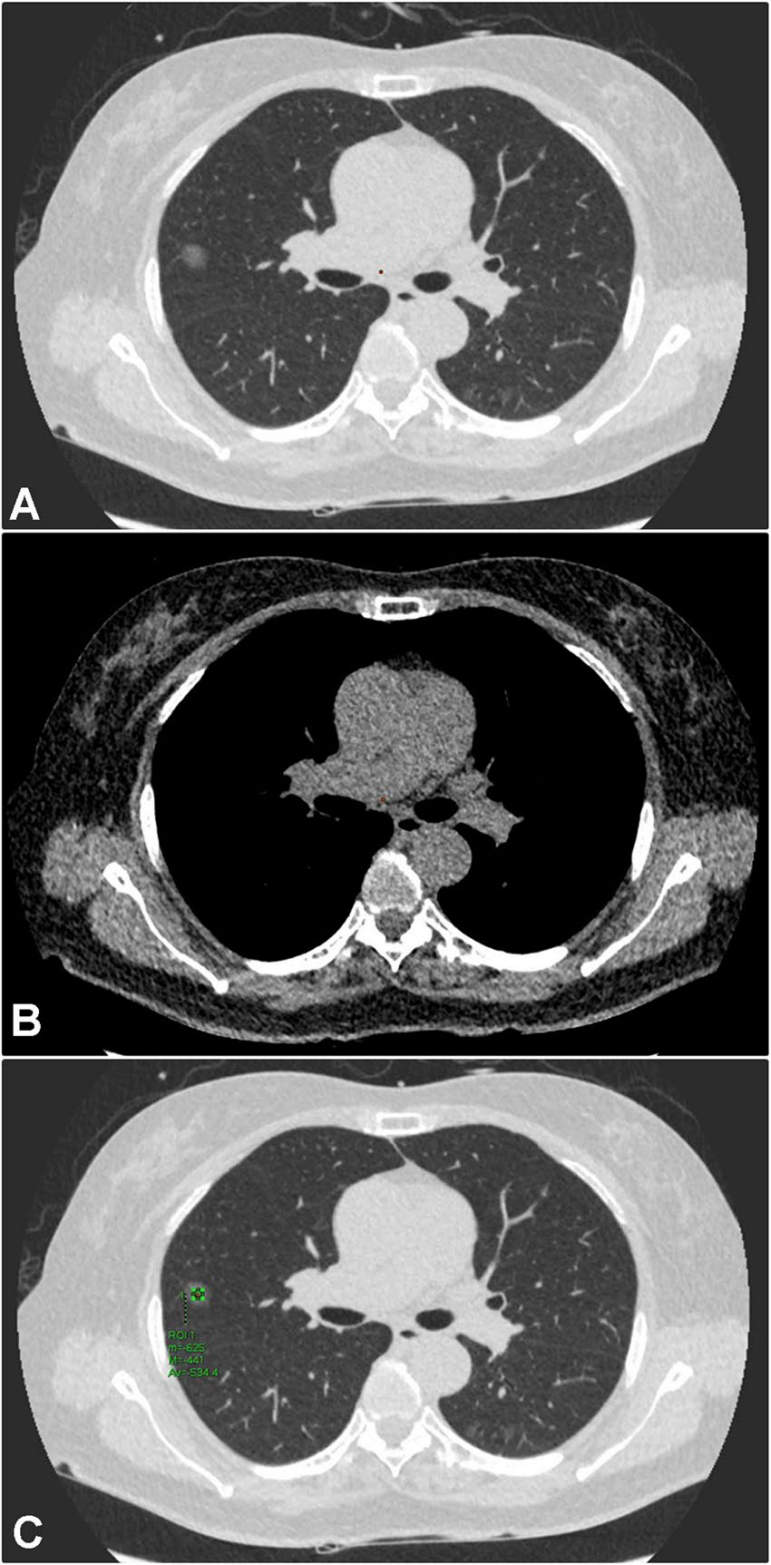
An example of pure GGN according to the SSN subclassification. A 60-year-old woman had a 1.3 cm pure GGN nodule in RML. The LDCT images showed homogenous groundglass opacities only when viewed on the lung window (Fig. 3A), but not seen on the mediastinal window (Fig. 3B). The average CT attenuation values (min, max and mean) expressed in HU were measured by placing a ROI of 15 mm^2^ on the lesion. In addition, to avoid placing the ROI box in or near the blood vessels could reduce the measurement errors (Fig. 3C). The patient underwent video-thoracoscopic wedge resection of RML. Further pathologic report demonstrated minimally invasive adenocarcinoma in RML. Abbreviations: GGN: groundglass nodule; SSN = subsolid nodule; RML = right middle lobe; ROI = region of interest; HU = Hounsfield unit.

**Figure 5:**
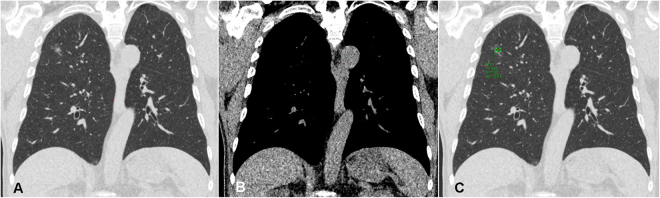
An example of heterogeneous GGN according to the SSN subclassification. A 66-year-old man had a 1.2 cm heterogeneous GGN nodule in RUL. The LDCT images showed heterogeneous groundglass opacities with focal solid component only when viewed on the lung window (Fig. 4A), but not seen on the mediastinal window (Fig. 4B). The average CT attenuation values (min, max and mean) expressed in HU were measured by placing an ROI of 15 mm^2^ on the lesion (Fig. 4C). In addition, to avoid placing the ROI box in or near the blood vessels could reduce the measurement errors. The patient underwent video-thoracoscopic wedge resection of RUL. Further pathologic report demonstrated invasive pulmonary adenocarcinoma in RUL. Abbreviations: GGN: groundglass nodule; SSN = subsolid nodule; RUL = right upper lobe; ROI = region of interest; HU = Hounsfield unit.

All scans were performed with a 16-slice CT (Somatom Sensation 16, Siemens Healthcare, Erlangen, Germany), a 64-slice CT (Aquilion 64; Toshiba Medical Systems), and 256-slice CT (Revolution CT, GE Healthcare, Milwaukee, USA) from the lung apex to the base without contrast enhancement. Images were reconstructed with a section thickness of 1–2.5 mm and displayed at a lung window width of 1600 HU, window level of −600 HU, and a mediastinal window width of 350 HU, with a window level of 35 HU. CT characteristics were retrospectively reviewed and analyzed by 2 experienced thoracic radiologists (FZW and MTW), with 9 and 30 years of experience in thoracic radiology, respectively. The imaging features for each nodule with pathologically proof were analyzed according to the following parameters: (1) lesion size, (2) solid part in a mediastinal window, (3) SSN subclassification into pure GGN, heterogeneous GGN (partly consolidated on lung windows), and part-solid nodules (with a mediastinal window solid component) according to the previous prospective study proposed by Kakinuma *et al*., (4) air-bronchogram (an example in Fig. 2), (5) abnormal cystic-like airspace (an example in Fig. 3), (6) Lung-RADS report of all SSNs, and (7) the attenuation values of pure and heterogeneous GGNs. Maximal diameters of the nodular lesion with solid components (in the maximal diameter when viewing using a mediastinal window setting) were measured on CT on axial images. The mean, minimum, and maximum CT attenuation expressed in HU was measured by placing a region of interest (ROI) of 15 mm^2^ on the lesion using a GE Advantage Server 2.0 (GE Healthcare). In addition, to avoid placing the ROI box in or near the blood vessels could reduce the measurement errors (examples in Figs 4 and 5).

On the basis of a random sample of 25 SSNs from a total of 141 SSNs evaluated by the two investigators (PAC and FZW). The interobserver agreements for SSN subclassification were high by kappa statistic (weighted Kappa = 0.875), whereas interobserver agreements for CT attenuation of GGNs (25 pure or heterogeneous GGNs) were high by intraclass correlation coefficient (ICC = 0.915; confidence interval CI 0.818–0.961). The previous studies also reported high interobserver agreement on the characteristic CT features such as nodule size, solid part size, and air-bronchogram^6,9^.

### Pathologic evaluation and report

All resected specimens were fixed in formalin and embedded in paraffin with haematoxylin and eosin staining for pathological diagnosis. The surgically resected SSNs specimens were histopathologically analyzed by two experienced thoracic pathologists according to the revised lung adenocarcinoma (IASLC/ATS/ERS) classification of 2011^5^. The discordant cases were subsequently discussed in a consensus meeting until a consensus was obtained.

### Statistical analysis

All statistical analyses were performed using SPSS 22.0 for Windows (SPSS Inc, Chicago, IL). Because all the continuous variables are normally distributed, Student t- test was used to test the differences between groups. Continuous variables are presented as mean ± standard deviation (SD). Differences in continuous variables between 2 groups were compared by the independent Student t test. Categorical variables were summarized as frequencies and percentages and compared using the chi-square test to examine differences in demographic characteristics. The Fisher exact chi-square test was used to analyze when the smallest expected value is less than 5.

Comparisons between groups stratified by SSN subclassification were determined by 1-way analysis of variance (ANOVA) and a linear regression model. ICCs were calculated for interobserver agreement for measurements of CT attenuation of GGNs. Kappa analysis was used for interobserver agreement regarding the novel SSN subclassification.

Univariate logistic regression was used to determine predictors for differentiating IPA lesions from pre-invasive/minimally invasive lesions. The optimal diagnostic threshold of parameters of CT features for differentiating IPA from pre-invasive/minimally invasive lesions was determined by use of c-statistics and the Youden index. ROC curves were plotted for parameters of CT features to confirm the optimal cut-off that differentiated the two groups. In addition, sensitivity, specificity, PPV, NPV, positive LR (LR+) and negative LR (LR−) were calculated to measure the overall accuracy of the multiple tests. The statistical significance for all tests was set at P < 0.05.

### Ethics Statement

The study protocol was approved by the institutional review board of Kaohsiung Veterans General Hospital (VGHKS16-CT12-11). Informed consent was waived due to the retrospective study design, and the study was performed in accordance with the Declaration of Helsinki.

### Data Availability

The datasets generated and/or analyzed during the current study are available from the corresponding author on reasonable request.

## Electronic supplementary material

Supplementary Information
