# Supplementary material for: Semiquantative Visual Assessment of Sub-solid Pulmonary Nodules ≦3 cm in Differentiation of Lung Adenocarcinoma Spectrum
Source: Sci Rep. 2017 Nov 17;7:15790. doi: 10.1038/s41598-017-16042-9 (PMC5694004; doi:10.1038/s41598-017-16042-9)

**Title**

Semiquantative Visual Assessment of Sub-solid Pulmonary Nodules ≦ 3 cm in

Differentiation of Lung Adenocarcinoma Spectrum

**Authors:**

Fu-Zong Wu^1,2,3,4,＊^, Po-An Chen^1,2^, Carol C, Wu^5^･Pei-Lun Kuo^1,2^, Shu-Ping Tsao^1,3,4^, Chu-Chun Chien^6,7^, En-Kuei Tang^8^, Ming-Ting Wu^1,2,＊^

**Author Affiliations:**

^1^ Department of Radiology, Kaohsiung Veterans General Hospital, Kaohsiung, Taiwan

^2^ Faculty of Medicine, School of Medicine, National Yang Ming University, Taipei,

Taiwan

^3^ Institute of Clinical Medicine, National Yang Ming University, Taipei, Taiwan

^4^ School of Medicine, College of Medicine, Kaohsiung Medical University

^5^ Department of Radiology, University of Texas MD Anderson Cancer Center, Houston, TX

^6^ Department of Pathology and Laboratory Medicine, Kaohsiung Veterans General Hospital, Taiwan

^7^ Department of Pathology, Kaohsiung Municipal Ta-Tung Hospital, Kaohsiung, Taiwan

^8^ Department of Surgery, Kaohsiung Veterans General Hospital, Kaohsiung, Taiwan

**Corresponding authors**

^＊^Correspondence and requests for materials should be addressed to F.Z.W. (email: [cmvwu1029@gmail.com](mailto:cmvwu1029@gmail.com)) or M.T.W. (email: [wu.mingting@gmail.com](mailto:wu.mingting@gmail.com))

**Grants:** This study was supported by Grants from Kaohsiung Veterans General

Hospital, VGHKS103-015, VGHKS104-048, VGHKS105-064, Taiwan, R.O.C.

| Supplement Table 1. CT finding of pre-invasive/minimally invasive lesions and IPA lesions in 62 part-solid nodules according to SSN subclassfication. | | | | |
| --- | --- | --- | --- | --- |
| Characteristic |  | Pre-invasive/minimally invasive (N=9) | IPA (N=53) | *P*-Value |
| Lesion size (mm) |  | 12.27±2.90 | 20.31±9.26 | 0.013^*^ |
| Solid part (mm) |  | 4.50±2.73 | 10.27±5.89 | 0.006^*^ |
| SSN subclassfication |  |  |  | N/A |
| Pure GGN |  | 0 | 0 |  |
| Heterogeneous GGN |  | 0 | 0 |  |
| Part-solid nodule |  | 9 | 53 |  |
| Air-bronchogram |  | 5 | 34 | 0.251^**^ |
| Cyst-like airspace |  | 0 | 4 | 0.525^***^ |
| ^*^ Using independent t-test for continuous variables; ^**^ Using Chi-square test for categorical variables; ^***^ Using Fisher´s exact test for categorical variables with expected frequencies < 5. | | | | |
| Abbreviations: GGN: groundglass nodule; IPA: invasive pulmonary adenocarcinoma; SSN: subsolid nodule; N/A: not applicable. | | | | |

| Supplement Table 2. CT imaging features of pre-invasive/minimally invasive lesions and IPA lesions in 141 subsolid nodules. | | | | |
| --- | --- | --- | --- | --- |
| Characteristics |  | Pre-invasive/minimally invasive (N=76) | IPA (N=65) | *P*-Value |
| Lesion size (mm) |  | 9.82±4.27 | 19.42±8.99 | <0.0001^*^ |
| Solid part (mm) |  | 0.57±1.73 | 8.37±6.66 | <0.0001^*^ |
| SSN subclassfication |  |  |  | <0.0001^***^ |
| Pure GGN |  | 53 | 4 |  |
| Heterogeneous GGN |  | 14 | 8 |  |
| Part-solid nodule |  | 9 | 54 |  |
| Air-bronchogram |  | 8 (12.30%) | 35 (46.05%) | < 0.0001^**^ |
| Cyst-like airspace |  | 0 (0 %) | 5 (6.57%) | 0.019 **^***^** |
| Abbreviations: GGN: groundglass nodule; SSN = subsolid nodule; IPA: invasive pulmonary adenocarcinoma. | | | | |
| ^*^ Using independent t-test for continuous variables; ^**^ Using Chi-square test for categorical variables; ^***^ Using Fisher´s exact test for categorical variables. | | | | |

| Supplement Table 3. Univariate logistic regression analysis for the different variables in distinguishing pre-invasive/minimally invasive lesions from IPA lesions in 141 subsolid nodules. | | | |
| --- | --- | --- | --- |
| Characteristics | Odds Ratio | 95% CI | P-Value |
| Age (year) | 1.073 | 1.031-1.115 | <0.0001 |
| Sex (female gender) | 0.997 | 0.425-2.342 | 0.995 |
| Lesion size (mm) | 1.389 | 1.242-1.553 | <0.0001 |
| Solid part (mm) | 1.682 | 1.416-1.998 | <0.0001 |
| SSN subclassfication | 9.007 | 4.878-16.631 | <0.0001 |
| Air-bronchogram | 9.917 | 4.113-23.909 | <0.0001 |
| Abbreviations: SSN = subsolid nodule; IPA: invasive pulmonary adenocarcinoma. | | | |

| Supplement Table 4. Univariate logistic regression analysis for the different variables in distinguishing pre-invasive/minimally invasive lesions from IPA lesions in 79 pure or heterogeneous GGNs. | | | |
| --- | --- | --- | --- |
| Characteristics | Odds ratio | 95% CI | P-value |
| Age (year) | 1.030 | 0.963-1.101 | 0.391 |
| Sex (female gender) | 1.204 | 0.235-6.171 | 0.824 |
| Lesion size (mm) | 1.218 | 1.075-1.380 | 0.002 |
| SSN subclassfication | 7.571 | 1.989-28.827 | 0.003 |
| Air-bronchogram | 1.939 | 0.185-20.373 | 0.581 |
| HU (mean) | 0.995 | 0.990-0.999 | 0.048 |
| Abbreviations: GGN: groundglass nodule; HU: Hounsfield unit; SSN: subsolid nodule; IPA: invasive pulmonary adenocarcinoma. | | | |

Supplement Figure 1

Graph shows ROC curves for CT parameters for the prediction of IPAs in 141 SSNs:

(A) lesion size ≧ 12 mm (B) solid part ≧ 3mm (C) part-solid nodule (D) air-bronchogram (+). Abbreviations: SSN = subsolid nodule; IPA = invasive pulmonary adenocarcinoma.


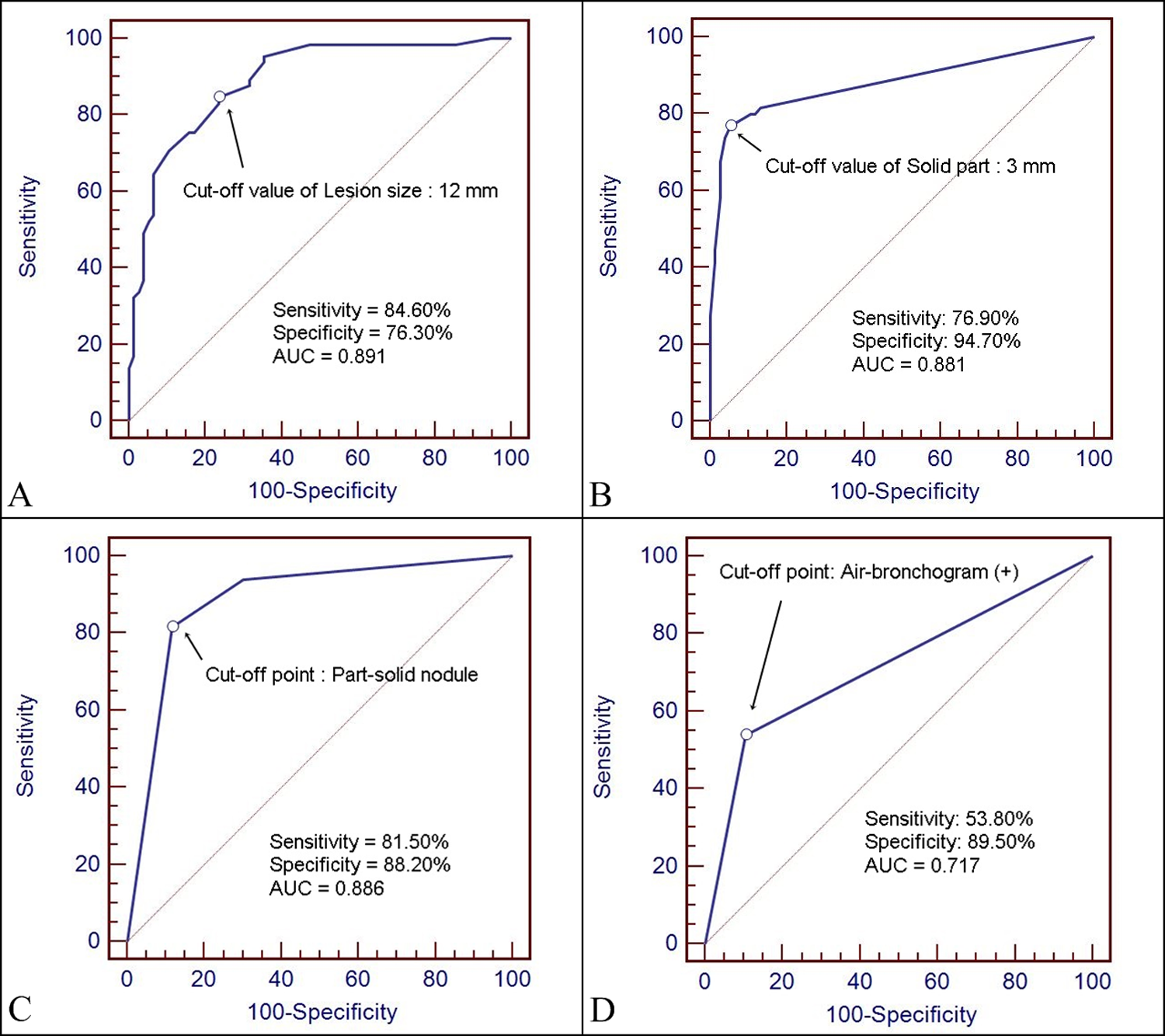


Supplement Figure 2

Graph shows ROC curves for CT parameters for the prediction of IPAs in 79 pure or heterogeneous GGNs : (A) lesion size ≧ 10 mm (B) heterogeneous GGN (C) HU attenuation ≧ -493. Abbreviations: GGN = groundglass nodule; IPA = invasive pulmonary adenocarcinoma; HU = Hounsfield unit.


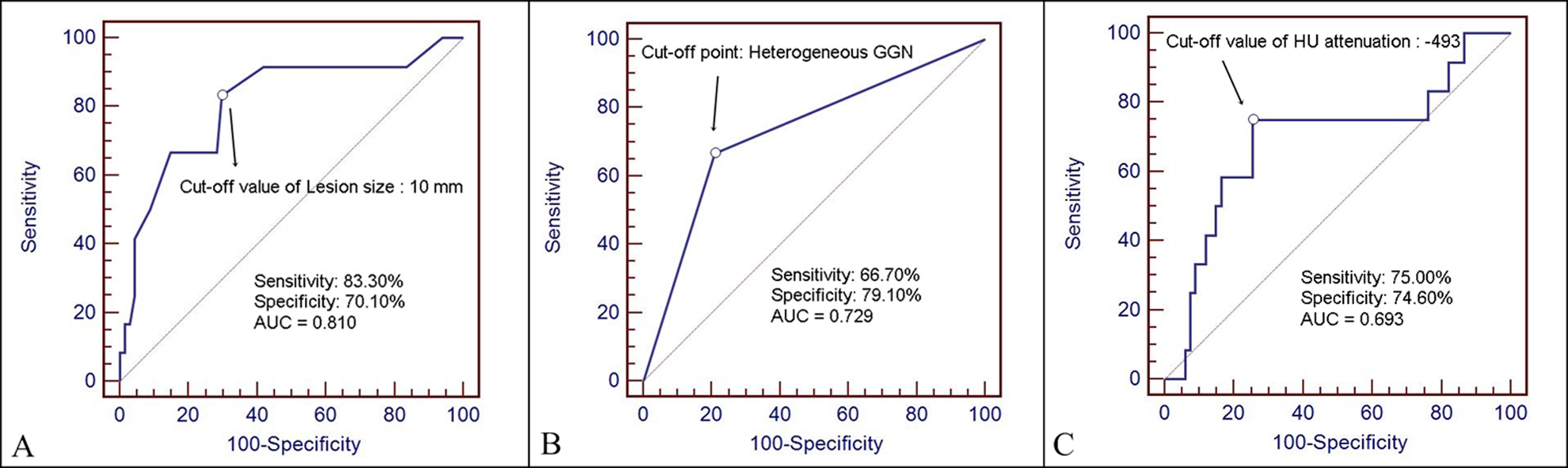

Supplement: Supplementary file 1 — Supplementary Information [file 41598_2017_16042_MOESM1_ESM.doc]
